# Perception and experience of academic Jordanian ophthalmologists with E-Learning for undergraduate course during the COVID-19 pandemic

**DOI:** 10.1016/j.amsu.2020.09.014

**Published:** 2020-09-11

**Authors:** Noor M. Alqudah, Hisham M Jammal, Omar Saleh, Yousef Khader, Nail Obeidat, Jumana Alqudah

**Affiliations:** a Division of Ophthalmology, Department of Special Surgery, Faculty of Medicine, Jordan University of Science and Technology, Jordan; b Department of Public Health, Community Medicine and Family Medicine, Faculty of Medicine, Jordan University of Science and Technology, Jordan; c Department of Obstetrics and Gynecology, Faculty of Medicine, Jordan University of Science and Technology, Jordan; d Department of Library and Information Science, Collage of Arts, University of Imam Abdulrahman Bin Faisal, Saudi Arabia

**Keywords:** E-Learning, COVID-19, Ophthalmology, Undergraduate, Jordan

## Abstract

**Background:**

Electronic-learning (e-Learning) is a form of education that utilizes information and communications technology to access online teaching and learning. This study aims to evaluate the e-Learning experience among Jordanian academic ophthalmologists during the coronavirus disease 2019 (COVID-19) pandemic.

**Material and methods:**

A cross-sectional survey was applied by using a questionnaire that was distributed among 23 academic ophthalmologists working at 6 medical schools in Jordan during the lockdown. The questionnaire included questions about the ophthalmologists' experience with e-Learning, advantages and disadvantages of e-Learning, interactions of medical students for the e-Learning and the expectations of e-Learning for the future.

**Results:**

A total of 22 out of 23 academic ophthalmologists responded. Flexibility of e-Learning to time and place was a major advantage (95.5%), whereas lack of skills was the main obstacle for e-Learning (77.3%). Nineteen participants (86.4%) were not satisfied with e-Learning as the sole method for undergraduate teaching. To improve the original on-campus two-week ophthalmology course, 12 (54.5%) suggested integrating e-Learning into the curriculum, 3 (13.6%) preferred extending the period of training, and 7 (31.8%) reported that their tight schedule does not allow for more tasks.

**Conclusion:**

The experience of e-Learning was positive. Most believed that e-Learning would have a prominent role in the future of medical education and proposed blended learning programs.

## Highlights

• The coronavirus pandemic has impacted medical education globally.
• E-Learning was not commonly used for medical education before COVID -19.
• E-Learning acceptance for undergraduate ophthalmology teaching in Jordanian universities was described in this study.
• E-Learning has a Promising future in medical education development.

## 1 Introduction

E-Learning is a type of learning or teaching platform that depends on electronic devices and technology instead of papers and classroom teaching [1]. Other terms that may be used to refer to this modern type of learning include virtual learning, web-based learning and computer-based learning [2]. E-Learning is not confined to online learning; it includes any form of digital communication used to deliver information. There are two main types of e-Learning: time-independent asynchronous type, where students study from downloadable courseware at their convenient time, and the synchronous type, where real-time online learning with the ability to interact and chat with students in live conferences is scheduled at set times [3]. As telecommunication methods are evolving every day, e-Learning is emerging as a modern technology that may have some advantages over traditional teaching methods including cost-effectiveness, regular updates, flexibility to time and place and accessibility to instructional information [4,5].

Many universities are incorporating e-Learning into their courses [6]. For medical education, the use of e-Learning is highly variable among countries. In US medical schools, e-Learning is mainly employed in teaching basic sciences [7]. However, for clinical sciences, education still heavily relies on clinical training, which enables student-patient interactions for history taking and examination. Where some studies have shown that e-Learning was equivalent to traditional learning in terms of acquiring knowledge and skills, other studies reported that blended learning, which integrates e-Learning with traditional methods, can be effective and may be well accepted by learners and instructors, predicting a shift in medical education in the future [8,9].

In Jordan, e-Learning is not well-utilized in the medical education and is being used mostly in the pre-clinical levels. On March 11, 2020, the World Health Organization (WHO) announced COVID-19 as a pandemic. Government authorities in Jordan activate a tight lockdown to combat the spread of the coronavirus, and all universities suspended on-campus teaching. E-Learning became the only choice to ensure the continuity of medical education in Jordan.

E-Learning may represent an opportunity to expand or tailor educational activities. Ophthalmology is part of the curriculum for undergraduate clinical training in medical schools with a variable duration for the course among different medical schools. Worldwide studies suggested that ophthalmology teaching at medical schools should be expanded or improved to enhance the graduates' knowledge and skills [10].

This study aims to evaluate the new experience of e-Learning among academic ophthalmologists in Jordan during the COVID-19 pandemic. We have assessed the ophthalmologists' perception about the effectiveness of e-Learning and their perception about the possibility of integrating e-Learning into the curriculum to achieve higher satisfaction for learners and instructors.

## 2 Material and Methods

After obtaining the ethical approval from the Institutional Review Board at our institution, a cross-sectional survey was conducted in April 2020 among 23 academic ophthalmologists in all 6 medical schools in Jordan. A structured questionnaire was developed using Google forms and was composed of 26 questions. An invitation to participate in the study was sent via email to the participants. The questionnaire included questions on the perception and experience of academic ophthalmologists with e-Learning during the COVID-19 outbreak, perceived advantages and disadvantages of e-Learning, attitude and interaction with medical students using e-Learning, perception of the present ophthalmology curriculum in Jordan, and the predictions of the e-Learning future in Jordan.

The questionnaire was anonymous to maintain the privacy and confidentiality of all information collected in the study. In addition, the participants' responses were kept blinded until all participants completed the questionnaire. Data were entered into a spreadsheet. Statistical analyses were performed using IBM SPSS Statistics Software (v.21), 2012. No statistical test was used due to the descriptive nature of the study. The sample size includes all the academic ophthalmologists.

This study was conducted according to the Strengthening the reporting of cohort studies in surgery (STROCSS) 2019 Guideline [11].

## 3 Results

### 3.1 Participants' characteristics

Out of 23 academic ophthalmologists distributed among 6 public universities in Jordan, 22 responded and participated in the online questionnaire. Six (27.3%) of them have been academic ophthalmologists for more than 10 years, 14 (63.6%) for 5 -10 years, and 2 (9.1%) for less than five years.

### 3.2 Perception and experience with e-learning

When asked about the use of e-Learning before the COVID-19 pandemic, 12 participants (54.5%) reported that they have never used it before in their academic career, 7 (31.8%) used it for some academic activities about half of the time, and 3 (13.6%) used it occasionally. During the COVID-19 lockdown, all participating ophthalmologists reported using e-Learning for all their academic activities. Fifteen participants (68.2%) started using it within the first week of the lockdown and the remaining 7 (31.8%) participants within 2–4 weeks.

Regarding arrangements with students, more than two-thirds of participants (n = 16; 72.7%) used both synchronous and asynchronous types of e-Learning, 5 (22.7%) participants used synchronous e-Learning only, and 1 participant (4.5%) used asynchronous e-Learning only.

The e-Learning platforms and applications used by the academic ophthalmologists are shown in Table 1.

**Table 1:** E-Learning platforms used by academic ophthalmologists during the COVID-19 lockdown in Jordan.

E-learning platform	N (%)
Zoom PowerPoint presentations with audio Microsoft Teams Discussion forums Recorded videos Others^a^	20 (90.9) 18 (81.8) 13 (59) 6 (27.3) 5 (22.7) 4 (12.8)

a Softwares including Facebook, WhatsApp, and Google Classroom.

Regarding perception of privacy and data protection while using these applications, 11 (50%) participants stated that they were not aware if e-Learning applications were secure or not, 10 (45.5%) thought these applications were secure, and 1 (4.5%) believed they were not secure. After starting the use of these e-Learning programs, 12 (54.5%) participants reported taking no precautions while using e-Learning software, and 10 (45.5%) participants followed the safety precautions recommended by their institutions' policy.

A total of 15 (68.2%) participants thought their technical skills were insufficient and chose to participate in courses arranged by the IT centers in their institutions to acquire or improve their e-Learning skills. Another 5 participants (22.7%) thought their skills were not sufficient and chose to enhance their skills by public online courses, and 2 (9.1%) believed that their skills were sufficient and that they didn't need any courses. For those who agreed to participate in courses to improve their e-Learning skills, 15 (75%) found that acquiring such skills was relatively easy, 5 (25%) reported that it was easy, and no one found it difficult.

Half of the participants (11, 50%) described their overall experience with e-learning as very good, 8 (36.4%) found it to be good, and 3 (13.6%) thought it was an excellent experience.

### 3.3 Perceived advantages and disadvantages of e-learning

Academic ophthalmologists were asked about the advantages, disadvantages, and limitations of e-Learning in Jordan. Their responses are shown in Table 2.

**Table 2:** Advantages and disadvantages of e-Learning as perceived by Jordanian academic ophthalmologists.

Advantages of e-Learning	Number of participants (%)
Not limited to time or place	21 (95.5)
Students are less shy to ask and interact	14 (63.6)
Students are more confident about their knowledge due to on-demand access to the material	4 (18.2)
Overcoming the circumstances of the current lockdown	1 (4.5)
**Disadvantages of e-Learning**	
Lack of personal interaction	14 (63.6)
The discomfort of teaching and learning without face to face mode	10 (45.5)
lack of clinical training and patient encounter	2 (9.1)
Highly subjective	1 (4.5)
**Limitations in e-Learning**	
Poor infrastructure	11 (50)
Shortage of e-Learning training courses	14 (63.6)
Poor e-Learning skills in students or teachers^a^	17 (77.3)
Speed of internet	13 (59.1)

a Including skills such as audio/video recording or editing, photo editing, and using applications for live conferencing.

### 3.4 Perceptions toward students' interaction and instructors' satisfaction with the learning process

For the frequency of their follow up with students, 10 (45.5%) of our participants said they checked the university's e-Learning website every day to answer student questions and suggestions, 9 participants (40.9%) said they checked it every 2-4 days, and 3 (13.6%) every 5-7 days. Generally, the ophthalmologists described the students' participation and interaction in the e-Learning process as good (11, 50%), very good (7, 31.8%) and fair (4, 18.2%).

As far as the clinical training in ophthalmology is concerned, 15 ophthalmologists (68.2%) reported that e-Learning programs could play a partial role in clinical training and 7 (31.8%) reported that none of the clinical part of training can be implemented electronically.

When asked if a two-week block dedicated for ophthalmology on-campus training course was enough and if e-Learning should be incorporated into it in the near future, 6 ophthalmologists (27.3%) considered the two weeks not to be enough. In addition, 12 (54.5%) thought that the original on-campus course may be improved by supplementing it by e-Learning programs, whereas 3 (13.6%) suggested extending the training period for more than 2 weeks. The remaining 7 participants (31.8%) preferred not to extend the original 2-week course due to their already tight schedule.

Lastly, when they were asked about their opinion in e-Learning as the sole method for ophthalmology training, 19 of the Jordanian academic ophthalmologists (86.4%) disagreed and 3 (13.6%) agreed.

### 3.5 Perception for the future impact of e-learning

Twenty (90.9%) participants believed that the lockdown gave them an unexpected opportunity to know more about e-Learning. Although the remaining 2 ophthalmologists (9.1%) said they have already been practicing some e-Learning from before the lockdown, they admit that they got to learn much more about it after the lockdown. For their future expectations, 21 (95.5%) participants predicted that e-Learning will have a greater role in education worldwide after the COVID-19 pandemic and only 1 (4.5%) thought it will not.

Regarding the participants’ personal willingness to use e-Learning after the lockdown is over, 12 participants (54.5%) showed their interest and 10 (45.5%) reported that they may use it sometimes. Moreover, 17 (77.3%) academic ophthalmologists were interested in conducting future e-Learning activities on a regular basis in cooperation with other departments of ophthalmology across the country, whereas 5 (22.7%) were not interested.

## 4 Discussion

In this study, we inquired through a questionnaire about the perception, experience, and future predictions related to e-Learning and its utilization among practicing academic ophthalmologists in Jordan during the COVID-19 pandemic.

In Jordan, the ophthalmology training course takes up about 2% of the full undergraduate clinical training program and lasts for two weeks during the fifth year in medical school. In the UK, Petrarca et al. reported that not all the desired ophthalmology topics could be covered in the short ophthalmology curriculums in their medical schools [10]. It has been shown that vital ocular signs that can be sight-threatening may be missed by general practitioners or junior doctors in emergency departments due to the limited ability to acquire all the necessary basic ophthalmology skills in the short period of training those practitioners had [12,13].

Almost one third of the academic ophthalmologists in Jordan believed that the ophthalmology course for undergraduates training is inadequately short and slightly more than half of them thought there could be a real benefit from adding e-Learning sessions to it.

In undergraduate ophthalmology training, certain clinical skills like direct ophthalmoscopy, pupil reactions, red reflex assessment, visual acuity assessment, eyelid eversion to check for foreign bodies, fluorescein staining of the cornea are essential [10,14]. The greatest challenge when using e-Learning is that students would have critically limited hands-on training in real-life scenarios, which is a key component in clinical education.

It was clear from the participants' responses that the COVID-19 lockdown provided them with a peculiar chance to use the e-Learning for undergraduates teaching. There was a consensus among the majority that their experience with e-Learning was positive and successful. What is more, almost two-thirds thought e-Learning could contribute to clinical training curriculums in the future.

One of the major advantages of e-Learning reported by the participants was the convenience and flexibility to time and place in the use of this platform. This is in keeping with findings from many studies reporting advantages of e-Learning including ease and fast access, the ability for quick revisions and updates of the material compared to textbooks and other modes of learning, the possibility of serving a large number of students with relatively low cost and the ability of e-Learning to potentially overcome differences between learners [15].

Most ophthalmologists in the present survey reported that the students' interactions during e-Learning sessions were good. Indeed, almost two third of participants believed that students were more expressive of their knowledge and more confident to interact with the instructor and with each other when using e-Learning applications. Some studies showed that the students' perception toward e-Learning was positive and students found it feasible and enjoyable [16,17]. On the other hand, two-thirds of our participants found that the lack of personal face-to-face interaction in e-Learning might make the clarifications, explanations, as well as interpretations less effective than the traditional method. This observation also echoed similar findings in previous studies [15].

Regarding limitations of e-Learning in Jordan, the majority of participants reported that poor technical skills in both the students and the instructors in using e-Learning techniques and the limited availability of e-Learning crash courses constitute a major obstacle in using this new platform. Not surprisingly, the majority of our ophthalmologists were willing to participate in courses for improving or acquiring e-Learning skills in the near future. It is known that unsatisfactory skills can be a serious downside in the application of e-Learning [18], for some learners or instructors may have excellent academic knowledge but may not necessarily have the skills to deliver that knowledge effectively [15]. Other hurdles for the utilization of e-Learning in Jordan, that have been reported by the majority of ophthalmologists, included the sparsity of high-speed internet spots and poor infrastructure, which could interfere with live conferencing.

The notion that e-Learning programs may sometime replace traditional on-campus teaching is highly doubtful. Studies did not demonstrate that e-learning was superior in delivering knowledge to the learners compared to conventional methods [19]. As an adjunct to conventional teaching, however, e-Learning may be highly useful and may have a significant impact on future medical students [20]. Studies have shown an acceptance of e-Learning in medical education when it's combined with the traditional methods [19,20]. Over half of the academic ophthalmologists in the present survey supported the use of e-Learning as a supplementary method to the traditional on-campus training.

COVID-19 pandemic has enabled the instructors all over the world to use e-Learning; it is advisable to consider using online learning for the future medical curricula [21].

Over time, e-Learning will mature, and information and communication technology will continue to develop. This will make it essential for both students and instructors to learn and develop their skills and stay updated in novel concepts and innovative techniques in the learning process and training programs.

Finally, limitations of this study include the relatively short period of time in which the survey participants have actually experienced e-Learning first-hand. There were only several weeks between the initiation of the COVID-19 lockdown and the participants responding to our survey. Additionally, the extraordinary economic and social circumstances that accompanied the lockdown coupled with the general feeling of uncertainty worldwide may have contributed, in both participants and the medical students they instruct, to a heightened level of personal stress and less enthusiasm to engage in medical research or surveys.

## 5 Conclusion

Despite all the challenges that may affect our e-Learning experience during the current global crisis, academic ophthalmologists in Jordan have found e-Learning to be constructive and practical and the students' interaction to be good. Notwithstanding, the majority agreed that it could not replace the traditional method of learning. Instead, they suggested integrating e-Learning programs to the original curriculum in order to enhance the academic performance. Flexibility to time and place was the most important advantage of e-Learning, and lacking e-Learning skills was the primary concern for the majority. Eventually, they agreed that e-Learning will play an influential role in the future of medical education.

## Ethics and patient consent

Institutional approval was obtained from the Institutional Review Board at Jordan University of Science and Technology. This study was conducted in accordance with the Declaration of Helsinki.

## Ethical approval

Institutional approval was obtained from the Institutional Review Board at Jordan University of Science and Technology (2020/238)

## Source of funding

No funding.

## Author contribution

All authors contributed significantly and in agreement with the content of the article. All authors were involved in project design, data collection, analysis, statistical analysis, data interpretation and writing the manuscript. All authors presented substantial contributions to the article and participated of correction and final approval of the version to be submitted.

## Trial registry number

Research registry: 5762.

## Guarantor

Dr. Noor M Alqudah.

## Provenance and peer review

Not commissioned, externally peer-reviewed.

## Declaration of competing interest

The authors declare that they have no competing interests.
